# Gene Cloning, Recombinant Expression, Characterization, and Molecular Modeling of the Glycolytic Enzyme Triosephosphate Isomerase from *Fusarium oxysporum*

**DOI:** 10.3390/microorganisms8010040

**Published:** 2019-12-24

**Authors:** Beatriz Hernández-Ochoa, Saúl Gómez-Manzo, Erick Alcaraz-Carmona, Hugo Serrano-Posada, Sara Centeno-Leija, Roberto Arreguin-Espinosa, Miguel Cuevas-Cruz, Abigail González-Valdez, José Alberto Mendoza-Espinoza, Marcelo Acosta Ramos, Leyda Cortés-Maldonado, Alba Mónica Montiel-González, Verónica Pérez de la Cruz, Luz María Rocha-Ramírez, Jaime Marcial-Quino, Edgar Sierra-Palacios

**Affiliations:** 1 Laboratorio de Inmunoquímica, Hospital Infantil de México Federico Gómez, Secretaría de Salud, Mexico City 06720, Mexico; beatrizhb_16@comunidad.unam.mx; 2 Laboratorio de Bioquímica Genética, Instituto Nacional de Pediatría, Secretaría de Salud, Mexico City 04530, Mexico; saulmanzo@ciencias.unam.mx (S.G.-M.); abelcob6@hotmail.com (E.A.-C.); leyda_cortez@hotmail.com (L.C.-M.); 3 Consejo Nacional de Ciencia y Tecnología (CONACYT), Laboratorio de Agrobiotecnología, Tecnoparque CLQ, Universidad de Colima, Carretera los Limones-Loma de Juárez, Colima 28629, Mexico; hjserranopo@conacyt.mx (H.S.-P.); scenteno0@ucol.mx (S.C.-L.); 4 Departamento de Química de Biomacromoléculas, Instituto de Química, Universidad Nacional Autónoma de Mexico, Mexico City 04510, Mexico; arrespin@unam.mx (R.A.-E.); miguelcuevascruz@ciencias.unam.mx (M.C.-C.); 5 Departamento de Biología Molecular y Biotecnología, Instituto de Investigaciones Biomédicas, Universidad Nacional Autónoma de México, Mexico City 04510, Mexico; abigaila@correo.biomedicas.unam.mx; 6 Colegio de Ciencias y Humanidades, Plantel Casa Libertad, Universidad Autónoma de la Ciudad de México, Mexico City 09620, Mexico; josealberto.mendoza@uacm.edu.mx; 7 Departamento de Parasitología Agrícola, Universidad Autónoma Chapingo, Maestría en Ciencias en Protección Vegetal, Carretera México-Texcoco km38.5, Chapingo, Estado de México, Texcoco 56230, Mexico; acostam14@gmail.com; 8 Departamento de Biotecnología, Universidad Autónoma Metropolitana-Unidad Iztapalapa, Mexico City 09340, Mexico; 9 Centro de Investigación en Genética y Ambiente, Universidad Autónoma de Tlaxcala, Aut. San Martín Texmelucan-Tlaxcala Km 10.5, San Felipe Ixtacuixtla, Tlaxcala 90120, Mexico; amonicamg@yahoo.com; 10 Departamento de Neuroquímica, Instituto Nacional de Neurología y Neurocirugía Manuel Velasco Suárez, S.S.A., Mexico City 14269, Mexico; veped@yahoo.com.mx; 11 Departamento de Infectología, Hospital Infantil de México Federico Gómez, Ciudad de Mexico 06720, Mexico; luzmrr7@yahoo.com.mx; 12 Consejo Nacional de Ciencia y Tecnología (CONACYT)-Instituto Nacional de Pediatría, Secretaría de Salud, Mexico City 04530, Mexico

**Keywords:** *Fusarium oxysporum*, triosephosphate isomerase, cloning, recombinant protein, biochemical characterization, homology modeling

## Abstract

Triosephosphate isomerase (TPI) is a glycolysis enzyme, which catalyzes the reversible isomerization between dihydroxyactetone-3-phosphate (DHAP) and glyceraldehyde-3-phosphate (GAP). In pathogenic organisms, TPI is essential to obtain the energy used to survive and infect. *Fusarium oxisporum* (Fox) is a fungus of biotechnological importance due to its pathogenicity in different organisms, that is why the relevance of also biochemically analyzing its TPI, being the first report of its kind in a *Fusarium*. Moreover, the kinetic characteristics or structural determinants related to its function remain unknown. Here, the *Tpi* gene from *F. oxysporum* was isolated, cloned, and overexpressed. The recombinant protein named FoxTPI was purified (97% purity) showing a molecular mass of 27 kDa, with optimal activity at pH 8.0 and and temperature of 37 °C. The values obtained for K_m_ and V_max_ using the substrate GAP were 0.47 ± 0.1 mM, and 5331 μmol min^−1^ mg^−1^, respectively. Furthemore, a protein structural modeling showed that FoxTPI has the classical topology of TPIs conserved in other organisms, including the catalytic residues conserved in the active site (Lys12, His94 and Glu164). Finally, when FoxTPI was analyzed with inhibitors, it was found that one of them inhibits its activity, which gives us the perspective of future studies and its potential use against this pathogen.

## 1. Introduction

*Fusarium* is a very diverse genus of filamentous fungi which is cosmopolitan and found in habitats such as soil, water, or associated with plants. Several of its species are saprophytes or endophytes, although pathogenic species that affect different mammals, including humans, have also been reported [1]. For that reason, the identification of species has been essential to analyze and study the biological behavior of the *Fusarium* species. In this sense, the identification of many species of *Fusarium* were classified according to their host associations and/or morphological characteristics, although the use of molecular techniques, such as PCR (amplifications of ribosomal/gene regions) and sequencing, has allowed a better differentiation and identification of these species [2,3]. Several of these fungal species are pathogenic mainly for plants, causing wilting and rotting of numerous agricultural crops. *Fusarium graminearum* and *F. oxysporum* are two of the species considered to be the most pathogenic [4]. In addition, in *F*. *oxysporum,* the so-called special forms (f. sp.) are of important relevance due to their high susceptibility to the host, causing various symptoms and damage to the plant. At least, 150 special forms have been found from isolates of *F*. *oxysporum*, depending on the type of host. In particular, *F*. *oxysporum* f. sp. *phaseoli* and *F*. *oxysporum* f. sp. *lycopersici* are species that affect bean and tomato crops, respectively, in several countries of Latin America, including Mexico, and have been an agricultural problem for several years [5,6]. Therefore, these fungi are considered a plague of several plant species, including cereals, tomatoes, beans, bananas, maize, and soybeans, with a great economic aftermath due to the low crop production [1,6,7,8].

Furthemore, *Fusarium* spp. also affects humans directly, causing the parasitosis called fusariosis, which can occur at different levels: superficial (keratitis or onychomycosis), locally invasive (sinusitis or intertrigo), and deep or disseminated infections, which occur particularly in immunocompromised patients [9,10]. It was even considered that *F. oxysporum* and *F. solani* were the most pathogenic species in humans, causing 70% of infections of this type [10].

Due to the impact of these fungi in the agricultural sector and their medical importance, because it is considered as an emerging and opportunistic group in humans [11], some researchers have studied them through different techniques to understand their physiology and, mainly, their pathogenic mechanisms to find alternatives for their control. The design and chemical synthesis of antifungal molecules have been used as a method to evaluate the inhibition of mycelial growth or to inhibit the activity of specific proteins, as evaluated in *F*. *graminearum* [12,13,14]. In recent years, the search for new molecules has been based on the analysis of specific targets such as proteins, of which intracellular signaling proteins, cytoskeleton, membrane, or enzymes that participate in the metabolism have been proposed for the treatment of pathogenic species [15].

The glycolysis pathway, despite being present in all organisms, some of its enzymes have been proposed as targets for drug design [16,17,18], because the sequence and structure of proteins differ between species. In this pathway, triosaphosphate isomerase (TPI; E.C. 5.3.1.1) is a highly conserved enzyme, important for energy formation and essential for the proper functioning of cells [19]. The main function of the TPI enzyme is to catalyze the interconversion of dihydroxyacetone phosphate into (D)-glyceraldehyde-3-phosphate; this process allows the two three-carbon molecules to continue being processed in the glycolytic pathway, since, without this reaction, there would be no net ATP production generated by the pathway [19]. Due to the relevance of this enzyme, some researchers have proposed the TPI as a potential target for the search (or synthesis) for new drugs [15,16,17,18], and even propose it as targets for the development of vaccines, of parasites that affect humans [16,20,21]. According to the aforementioned, it was in our interest to study the TPI of the fungus identified as *F. oxysporum*, since, despite being a pathogenic fungus of plants and/or humans, the analysis of biochemical characterization of this or another enzyme of the glycolysis had not been performed previously.

Then, based on the background of studies conducted for the TPI in different pathogens, we decided to perform the isolation of the *Tpi* gene from a wild species of *Fusarium* collected from a bean crop. To achieve our objective, the gene was obtained from cDNA, cloned into a vector and overexpressed in *Escherichia coli* in order to obtain enough recombinant protein to perform all biochemical assays. The results of the kinetic and structural parameters, the prediction of the model as well as the use of inhibitory molecules made in this study allowed us to determine that FoxTPI shows similar catalytic and structural properties to that of other microorganisms and that this enzyme, to be inhibited in the same way, can be proposed as a target for this fungus that is of biotechnological interest in areas such as veterinary, agricultural, and medical.

This knowledge will contribute to a better understanding of a protein that can play an essential role in the metabolism of *Fusarium*, which would allow the implementation of other approaches to control these pathogens through the analysis of existing molecules or the design and synthesis of new molecules.

## 2. Materials and Methods

### 2.1. Source of Fungal Strain

The *Fusarium* fungus used in this study was isolated from a bean culture and was molecularly identified by the sequence of PCR amplified fragments of the large subunit (LSU) of the nuclear ribosomal RNA (rRNA) gene [22]. The primers LSU Fw and Rv used to amplify this region are shown in the Supplementary Materials Table S1. The fungus was grown in Petri dishes containing potato dextrose agar (PDA) medium to produce spores, which were preserved in 40% glycerol (*v*/*v*) and placed at −20 °C.

### 2.2. Nucleic Acids Extraction, RT-PCR, and Cloning

DNA and RNA were isolated from 200 mg of mycelium grown in potato dextrose broth, frozen and pulverized in liquid nitrogen. The DNA was obtained using the classic phenol:chloroform method according to the protocol of Sambrook and Russel [23], whereas RNA was extracted using the Trizol reagent (Invitrogen, Carlsbad, CA, USA), following the manufacturer’s protocol. The purified RNA samples were placed at −80 °C until use.

Then, the total RNA extracted (1 µg) was treated with DNAse I and subsequently used for the synthesis of cDNA, in a reaction that contained dNTP’s Mix (10 mM), oligo(dT)_18_ primers, and *Revertaid* reverse transcriptase (Thermo Scientific, Waltham, MA, USA). The synthesis conditions were those established for transcriptase.

The full length of the *Tpi* gene was amplified by conventional PCR using degenerate oligonucleotides. These primers were designed from sequences deposited in Fungidb (http://fungidb.org/fungidb/) [24] using different species of *Fusarium* (Figure S1). The primers were named Fw Tpi and Rv Tpi, to which were added the site *Nde*I and *Bam*HI, respectively; the sequence of the primers is reported in Supplementary Materials Table S1. The PCR reaction was made as follows: 100 ng of template (DNA or cDNA), 200 ng of primer, 10 mM dNTP’s mixture, 1× PCR buffer and 1 U of Phusion^®^ High Fidelity DNA polymerase (Thermo Scientific). The PCR conditions were used according to the Polymerase protocol and programmed in a T100^TM^ Thermal cycler (Bio-Rad, Hercules, CA, USA). The obtained amplicons were separated using 1% agarose gel electrophoresis, stained with GelRed (Nucleic Acid Gel, Biotium, Fremont, Alameda, CA, USA), and visualized on a MultiDoc-It equipment (UVP, Upland, San Bernardino, CA, USA). Then, the PCR product was ligated into a vector of CloneJET PCR Cloning Kit (Thermo Scientific) following the indicated protocol. The resulting plasmid was named pJET-*Tpi* and was transformed into *Escherichia coli* TOP 10F’ cells. The plasmid DNA was extracted using the GeneJET Plasmid Miniprep Kit (Thermo Scientific), to sequence and verify the fidelity of the FoxTpi gene. The primers of the pJET vector were used for sequencing, also listed in Supplementary Material (Table S1).

**Figure 1:**
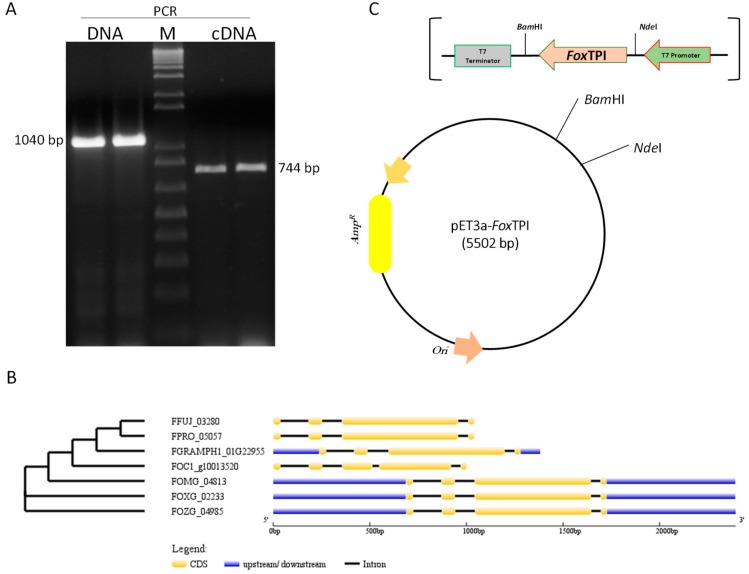
Isolation, analysis, and cloning of the *FoxTpi* gene. (**A**) amplification of the gene from DNA and cDNA by PCR and RT-PCR, respectively, using degenerate primers. (M) An amount of 1 kb Plus DNA Ladder Marker (Invitrogen); (**B**) structural and phylogenetic analysis of *Tpi* genes among *Fusarium* species; graphic representation of exon–intron structures displayed using GSDS. FFUJ_03280 (*F*. *fujikuroi* IMI 58289), FOMG_04813 (*F*. *oxysporum* f. sp. *melonis* 26406), FGRAMPH1_01G22955 (*F*. *graminearum* PH-1), FOXG_02233 (*F*. *oxysporum* f. sp. *lycopersici* 4287), FOC1_g10013520 (*F*. *oxysporum* f. sp. *cubense race* 1), FPRO_05057 (*F*. *proliferatum* ET1), FOZG_04985 (*F. oxysporum Fo47*); (**C**) vector constructed for the expression of the recombinant FoxTPI protein, named pET3a-HisTEV-FoxTPI, carrying the *FoxTpi* gene and regulating by T7 promoter.

After, the vector pJET-*Tpi* was digested with the enzymes *Bam*HI and *Nde*I to release the region of the *Tpi* gene and subclone it into the plasmid pET3a-HISTEVP, this vector was named pET3a-HISTEVP-*Fox*TPI (Figure 1). This plasmid contains a nucleotide region encoding the 6xHis tag and a cutting sequence for the tobacco kinase enterokinase proteinase (TEVP), which is bound at the 5′ end of the *Tpi* gene. Then, pET3a-HISTEVP-*Fox*TPI was transformed into *E*. *coli* TOP10F’ cells and the selection of the transformants was carried out in plates with Luria Bertani (LB) medium and ampicillin (*Amp^R^*). Finally, pET-3a-HISTEVP-*Fox*TPI was transformed into *E*. *coli* BL21 (DE3) pLyS for the expression of the recombinant FoxTPI protein.

### 2.3. Aligment of FoxTPI Protein

A total of 21 amino acid sequences were obtained from the National Center for Biotechnology Information (NCBI) [25] and the sequences were selected from the Protein Data Bank proteins (PDB) database, and a multiple sequence alignment (MAS) of TPIs amino acid sequences was performed with the Jalview (v.2.11.0) [26] software (University of Dundee, Dundee, UK) using ClustalW with the parameter by default.

### 2.4. Expression and Purification

*E. coli* BL21(DE3)pLyS containing the plasmid (pET-3a-HISTEVP-*Fox*TPI) was grown at 37 °C in LB medium plus 100 mg/L ampicillin, and the expression was induced with isopropyl β-d-galactopyranoside (IPTG) (0.4 mM) for 16 h at 30 °C. The culture was centrifuged, and the pellet formed was resuspended in lysis buffer, pH 8.0 (50 mM Tris, 50 mM NaCl, 5 mM β-mercaptoethanol, and 1 mM phenylmethylsulfonyl fluoride (PMSF)), to disrupt the cells by sonication. Then, the lysed cells were centrifuged for 20 min (15,000× *g* at 4 °C), and the clear supernatant was named as crude extract, which was used for the purification of FoxTPI protein. Next, the crude extract was loaded to a Ni Sepharose^®^ high performance column (GE Healthcare, Chicago, IL, USA) pre-equilibrated with binding buffer pH 8.0 (50 mM Tris, 150 mM NaCl). Then, the column was washed with five column volumes of washing buffer (binding buffer plus 150 mM NaCl, 60 mM imidazole, and 2 mM DTT). Thereafter, the bound protein was eluted with the same binding buffer plus imidazole (250 mM) and DTT (2 mM) [27]. Imidazole was eliminated from the sample by five consecutive dilutions binding buffer and concentrated using a microcon-30 kDa centrifugal filter unit (Millipore, Burlington, MA, USA). The 6xHis tag region present in the N-terminus of the FoxTPI protein was eliminated by incubating with the tobacco virus protease (TEVP). The purified protein was preserved in glycerol (50% *v*/*v*) and stored at −70 °C. The protein extracts obtained in each purification step, as well as the purified FoxTPI protein were analyzed in 10% SDS-PAGE gels [28] and stained with 0.05% colloidal Coomassie (R-250). Protein quantification was performed by Lowry et al. [29] using bovine serum albumin (BSA) as a standard.

### 2.5. Characterization of Functional of FoxTPI

#### 2.5.1. Oligomeric Status of the Protein

The determination of the oligomeric state of FoxTPI was performed by gel filtration chromatography (GFC) using a Sephacryl 100 (16/60) column (GE Healthcare, Amersham Pl, Little Chalfont, UK) coupled to an AKTA pure fast protein liquid chromatography (FPLC) equipment (GE Healthcare, Chicago, IL, USA). The column was equilibrated with 50 mM Tris buffer (pH 8.0), then the protein was loaded at a flow rate of 0.5 mL/min in equilibrium buffer. To elute the protein, the same buffer was used as mobile phase and protein detection was carried out following its absorbance signal at 280 nm. Moreover, the column calibration was carried out with gel filtration standard # 151-1901 (Bio-Rad), which contains thyroglobulin (bovine), γ-globulin (bovine), ovalbumin (chicken), myoglobin (equine), and vitamin B12. The migration of the FoxTPI protein was compared to the standard molecular weight (MW) marker.

#### 2.5.2. Effect of pH on Activity

To evaluate the effect of pH on the activity of FoxTPI, we measured the activity through an assay coupled using a pH range of 6.0 to 10.0, and the optimum pH for FoxTPI activity was determined. The buffers used were 2-(N-morpholino)ethanesulfonic acid (MES), pH 6.0–6.75; 4-(2-hydroxyethyl)-1-piperazineethanesulfonic acid (HEPES), pH 6.75–8.0, Tris, pH 8.0–9.0, and glycine, pH 9.0–10; all buffers were used at 50 mM. FoxTPI activity was determined as previously reported by Hernández-Ochoa et al. [18]. The non-enzymatic oxidation of reduced nicotinamide adenine dinucleotide (NADH) in the coupled assay was determined at each pH value and subtracted from the experimental points. The enzyme activity determined at opimun pH was set to 100%. The experiment was performed in triplicate.

#### 2.5.3. Effect of Temperature on Activity

The effect of the temperature on the FoxTPI activity was analyzed by thermal inactivation analysis. The FoxTIM protein was adjusted to 0.2 mg/mL in buffer TE (0.1 M Trietanolamine plus 10 mM ethylenediaminetetraacetic acid (EDTA) and incubated in a temperature gradient ranging from 10 to 60 °C for 20 min [30]. The measurements of activity at each temperature served to determine the residual activity of FoxTPI, which were expressed as a percentage of activity of the same enzyme incubated at 37 °C. All thermal inactivation trials were performed in triplicate. The enzyme activity before pre-incubation was adjusted to 100%.

#### 2.5.4. Determination of Steady-State Kinetic Parameters

FoxTPI enzyme activity was measured using a coupling enzyme assay at 25 °C as described by Hernandez-Ochoa et al. [18], following the conversion of glyceraldehyde-3-phosphate (GAP) to dihydroxyacetone phosphate using α-glycerolphosphate dehydrogenase (α-GDH). The reaction mixture in a final volume of 1 mL contained GAP (1 mM), NADH (0.2 mM), and 0.9 units/mL of α-GDH in TE buffer (pH 7.4) and with 5 ng/mL of FoxTPI. The NADH oxidation was determined at 340 nm. The experimental steady-state kinetic parameters of the FoxTPI enzyme were obtained by measuring the initial velocity data by varying the GAP concentration (0 to 3 mM). The results of the initial velocity obtained for each concentration were adjusted to the Michaelis-Menten equation by nonlinear regression calculations [18], which allowed us to obtain the kinetic parameters of steady state, K_m_, k_cat_ and V_max_. The experiment was performed in triplicate.

### 2.6. Evaluation of FoxTPI Protein Stability

#### 2.6.1. Thermal Stability

The effect of temperature on the global stability of the FoxTPI protein was evaluated by thermal denaturation by circular dichroism (CD). The protein was adjusted to 0.4 mg/mL with buffer P (0.05 M potassium phosphate buffer pH 7.4), and the thermal denaturation was monitored at 222 nm in temperature scans ranging from 20 to 90 °C, increasing at a rate of 1 °C/2.5 min. The assay was performed in a spectropolarimeter (Jasco J-8190 ^®^, Easton, MD, USA). It is considered that the temperature at which 50% of the folded and 50% is unfolded of the protein, it is expressed as the melting temperature (T_m_) and was obtained with the Boltzmann adjustment equation of the Origin program (version 8, 2016) [31]. The spectrum of the blank was subtracted from those that contained the FoxTPI enzyme. This assay was also performed in triplicate.

#### 2.6.2. Stability of FoxTPI in the Presence of Guanidine Hydrochloride (Gdn-HCl)

The stability of FoxTPI enzyme was performed in the absence and presence of chaotropic agents as Gdn-HCl. The purified FoxTPI protein was adjusted to 0.2 mg/mL of enzyme concentration and incubated at 37 °C for 2 h, with different concentrations of Gdn-HCl, from 0 to 4 M. Then, the residual activity of FoxTPI was determinated as previously indicated and expressed as a percentage of the activity. The experiments were performed in triplicate.

### 2.7. Spectroscopic Characterization of the FoxTPI Protein

#### 2.7.1. Circular Dichroism (CD) Analysis

The secondary structure of FoxTPI was evaluated by CD using a spectropolarimeter (Jasco J-8190 ^®^, Easton, MD, USA) as reported by Gómez-Manzo et al. [31]. A quartz cuvette and a protein concentration of 0.4 mg/mL of purified FoxTPI protein was suspended in buffer P. A far UV–CD spectrum of the FoxTPI protein was obtained from 190 to 260 nm with a scanning speed of 50 nm min^−1^. Spectra of blanks were subtracted from those that contained the protein. The curves were plotted using the Origin program. The assays were performed in duplicate at 37 °C.

#### 2.7.2. Structural Analysis through Intrinsic Fluorescence

The intrinsic fluorescence emitted from tryptophan residues present in proteins has been employed as a tool to determine the structural analysis of them [26,27,28,29,30,31,32,33,34]. The fluorescence spectrum of the FoxTPI was obtained at 25 °C, in a range between 310 and 500 nm using a PerkinElmer LS-55 equipment (PerkinElmer, Wellesley, MA, USA). In particular, FoxTPI presents four tryptophans residues per monomero, and the intrinsic fluorescence was monitored after excitation at 295 nm in buffer P at a protein concentration of 0.1 mg/mL in a quartz cuvette with a path length of 1 cm. Tryptophan intrinsic fluorescence was also monitored in presence of different Gdh-HCl concentrations (from 0 to 4 M) incubated at 37 °C for 2 h. In both trials, the data of the final spectra were corrected by subtracting the blank sample (without protein). Three independent experiments were performed.

### 2.8. Bioinformatics and Homology Modeling of FoxTPI

The predicted and annotated sequence of FoxTPI was used to perform a BLAST protein data bank (PDB) using a target database of 3D structures. The homology model of the full-length FoxTPI enzyme was built on the Swiss-Model server [35] using the crystallographic structure of the TPI from *Eukaryotes* (TIM63; PDB entry 6NEE; sequence identity: 68.5%) [36] as a template for calculations. The best model was selected according to the global model quality estimation (GMQE), quaternary structure quality estimate (QSQE), and qualitative model energy analysis (QMEAN) statistical parameters and subjected to energy minimization using the YASARA software [37]. The homology model of FoxTPI was validated using MolProbity [38] and structural analysis was performed manually using Coot [39]. The FoxTPI model (Supplementary Materials FoxTPI_Minimized.PDB) was also aligned with a crystal structure containing glyceraldehyde 3-phosphate (G3P; PDB entry 3UWU) [40] using Coot [39]. This structural alignment was used to position the G3P substrate into the FoxTPI model and then re-minimized using the YASARA software (Institute for Molecular biology, Biochemistry and Microbiology, IMBM, University of Graz, Graz, Austria; Center for Molecular and Biomolecular Informatics, CMBI, University of Nijmegen, Nijmegen, the Netherlands) [37]. Graphical representations were made using CCP4mg version 2.10.10 software (University of York, Heslington, York, UK) [41].

### 2.9. Assay Inactivation

In order to determine if FoxTPI could be inactivated by the derivatization of its Cys residues, and be able to propose it as a target for the rational design of drugs, three compounds called BHO1, BHO2, and BHO3 previously reported by Hernández-Ochoa et al. [18] were used. The authors used these compounds to inhibit the TPI of *Giardia lamblia* by derivatizing their cysteines (Cys). In this way, the FoxTPI was incubated for 2 h in the presence of compounds at concentration ranging from 50 to 500 µM in TE buffer at 37 °C, for assays FoxTPI (0.2 mg/mL), were incubated in a final reaction volume of 50 µL, the stock solutions of compounds were prepared at the time of testing, using dimethyl sulfoxide (DMSO) as solvent, the DMSO concentration during incubation was maintained to 5%, the concentration at which the activity of the enzyme is not affected. At the end of the incubation time, an aliquot was withdrawn and diluted (200 times), and the residual activity was measured spectrophotometrically at 340 nm, and the activity of the enzyme incubated without compounds was set as 100% activity.

## 3. Results and Discussion

*F. oxysporum* is a fungus that lives mainly in the soil, which leads to being one of the pathogenic fungi that affect various crops of agricultural importance in the world, generally causing wilting of the plant and root rot. This fungus has a wide host range, including, lettuce, leeks, tomatoes, onions, peppers, beans, spinach, peas, watermelons, strawberries, and bananas. In fact, *F*. *oxysporum* was named as the fifth most important plant pathogenic fungus [4]. Therefore, it is relevant to investigate for alternatives against this type of pathogens; one strategy is the search of targets; in this sense, proteins in recent years have been used as a model for the design of specific molecules, mainly of those that are essential in the growth or metabolism of the pathogen, and that present differences in sequence and structure with the host’s protein.

One enzyme studied and well characterized in different organisms is the triosephosphate isomerase (*TPI*); this is the fifth enzyme of the glycolytic pathway, which catalyzes the reversible isomerization between dihydroxyactetone-3-phosphate (DHAP) and glyceraldehyde-3-phosphate (GAP) [17]. In most organisms, including pathogens, this enzyme is essential to obtain the energy used to survive and infect. It is also relevant to know its pathogenic implications and the few compounds reported and used for its treatment. However, the isolation of this key enzyme has not been reported to *Fusarium* genus. Moreover, the kinetic characteristics or structural determinants related to its function remain unknown, which could be utilized for biotechnological purposes for the design of molecules in the medical and agricultural area.

### 3.1. Sequence Analysis and Cloning of FoxTpi Gene

Through the use of degenerate primers designed from *Tpi* sequences deposited in FungiDB (http://fungidb.org/fungidb/), a 1040 bp fragment was amplified by PCR; from the extracted RNA, we obtained fragments of approximately 744 bp by RT-PCR (Figure 1A). Both fragments obtained from DNA and RNA were ligated into the pJET 1.2 vector and confirmed by sequencing. The sequences obtained from the amplified fragments from DNA and RNA were analyzed by blast nucleotide (BLASTn) [25], and both sequences showed 100% similarity to the sequence of the *Tpi* gene of *F*. *oxisporum* f. sp. *lycopersisi* (No. Accsession XP_018235652.1) deposited in the GenBank. Considering the differences in length between the fragments, the exon–intron regions of the *FoxTpi* gene were analyzed according to other sequences of reported *Fusarium* strains. According to the analysis of the sequences of the TPI gene of different *Fusarium* species, it was observed that most have three introns, and in particular in *F*. *oxisporum* (our study strain), the length of these is 146, 101, and 49 bp, similar to strains *F*. *oxysporum* Fo47 (FOZG_04985) and *F*. *oxysporum* f. sp. *melonis* (FOMG_04813) with the exception of *F*. *oxysporum* f. sp. cuban strain 1 (FOC1_g10013520), which has four introns, and, despite being genes that encode the same protein, also have phylogenetic differences between them (Figure 1B). Then, the *FoxTpi* cDNA was subcloned into the pET3a-HisTEVP vector to express the protein in *E*. *coli*. The *FoxTpi* gene (at the 5′ end) contains the 6xHis tag region present in the vector (Figure 1C).

### 3.2. Alignment of the FoxTPI Protein

**Figure 2:**
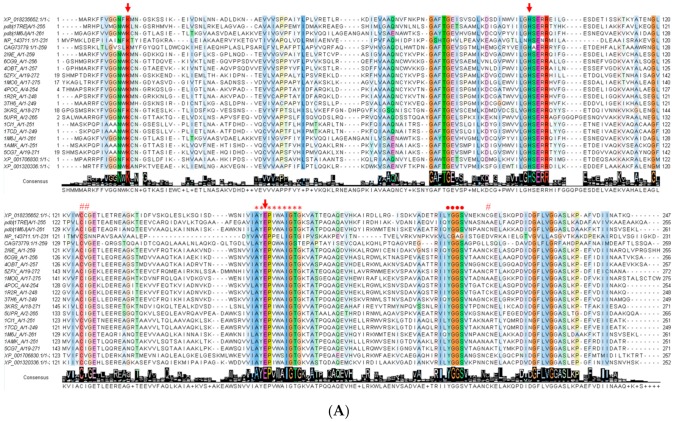

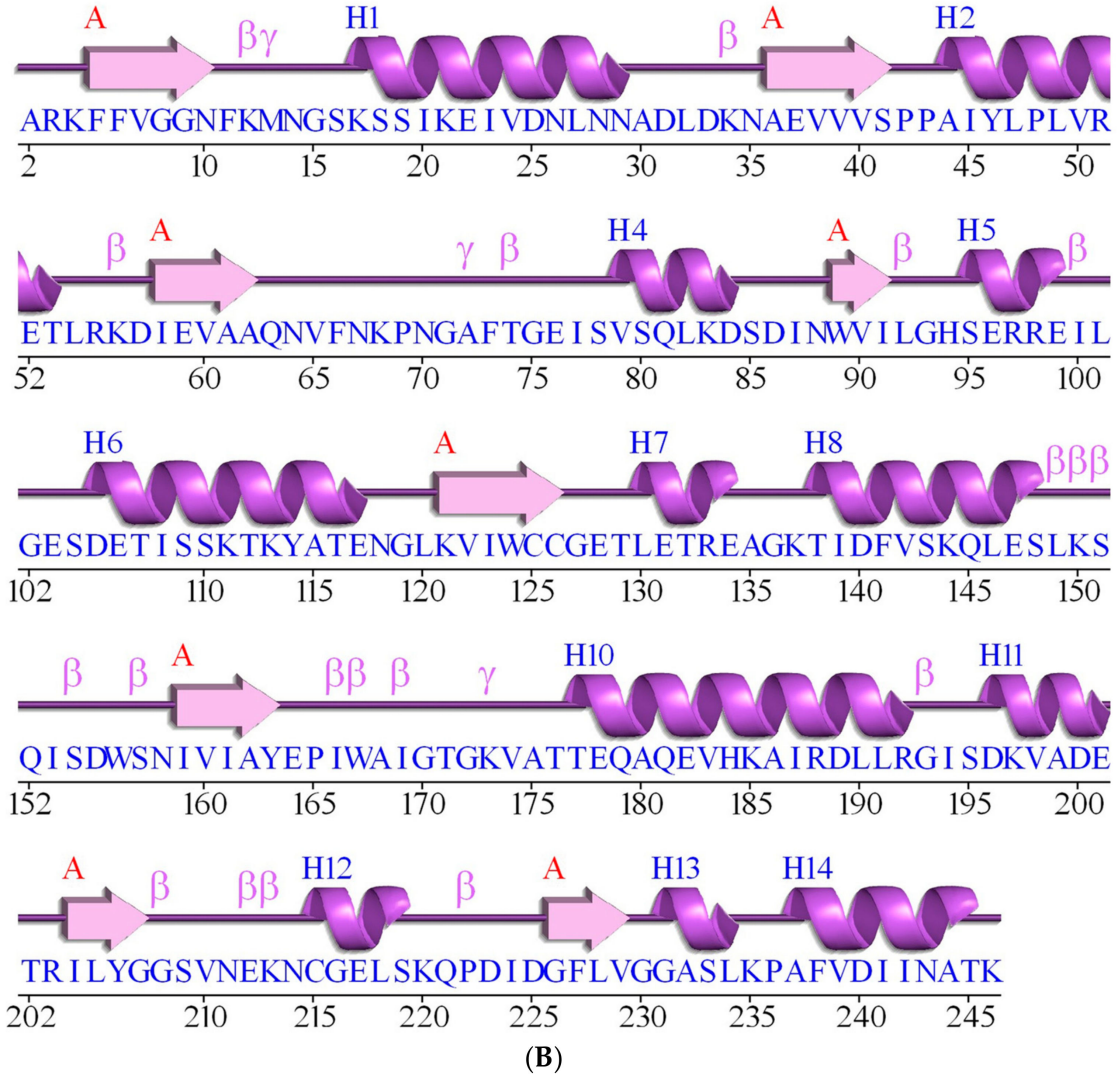
Bioinformatic analysis of FoxTPI with other triosephosphate isomerases (TPIs). (A) Sequence alignment of 21 TPIs and precition of secondary structure. The sequence alignment of TPIs from XP_018235652.1 (*Fusarium oxysporum* f. sp. *lycopersici* 4287), 1TRE (*Escherichia coli*), 1M6J (*Entamoeba histolytica*), NP_143711.1 (*Pyrococcus horikoshii* OT3), CAG73779.1 (*Pectobacterium atrosepticum* SCRI1043), 2I9E (*Tenebrio molitor*), 6CG9 (*Zea mays*), 4OBT (*Arabidopsis thaliana*), 5ZFX (*Opisthorchis viverrini*), 1MO0 (*Caenorhabditis elegans*), 4POC (Wild Type human), 1R2R, 3TH6 (*Rhipicephalus microplus*), 3KRS (*Cryptosporidium Parvum*), 5UPR (*Toxoplasma gondii*), 1CI1 (*Trypanosoma cruzi*), 1TCD (*Trypanosoma cruzi*), 1AMK (*Leishmania mexicana*), 5CG7 (*Leishmania siamensis*), XP_001706830.1 (*Giardia lamblia* ATCC 50803), XP_001320336.1 (*Trichomona vaginalis* G3) was performed using ClustalW (1.81) [29]. The uniform and solid colors indicate conserved amino acid in the sequences. Semi-colored and uncoloured, represent less conserved or non-conserved amino acids, respectively. The active site amino acid residues (↓), Cys residues (#), and TPI consensus signature (*) and other conserved regions of catalytic loop (●) of FoxTPI; (**B**) the secondary structure of the FoxTPI was predicted with PDBsum [43]. The numbers indicate the corresponding amino acids residues of the FoxTPI protein and the secondary structure elements are shown as α-helices and β-sheets.

The *FoxTpi* gene encodes a protein of 247 amino acids and has a calculated molecular mass of 27,090 Da and a theoretical isoelectric point (pI) of 5.29, which was determined with the ProtParam tool from the Expasy online program [42]. To know the amino acid conservation of the FoxTPI enzyme, the nucleotide sequence obtained was translated to protein to compare it with different proteins of the TPI family. Considering that there are deposited crystallographic structures of TPIs of some organisms including parasites of medical importance, we chose 21 sequences from the Protein Data Bank proteins (PDB) database to perform the alignment. Multiple sequence alignment of the FoxTPI proteins revealed fragment corresponds in some regions and revealed a 17% to 80% sequence identity with respect to the TPIs analyzed from other organisms (Figure 2A). As seen in Figure 2A, the FoxTPI amino acid sequence contains the motif AY**E**PIWAIGTG (*), its highly conserved motif is nearly the same as the reported TPI consensus signature [AVG]-[YLV]-**E**-P-[LIVMEPKST]-[WYEAS]-[SAL]-[IV]-[GN]-[TEKDVS]-[GKNAD], where **E** represents the active site residue (PROSITE access number: PS00171). It was also found that the active site residues (Lys12, His94, and Glu164) (↓) are conserved in the FoxTPI sequence. Furthermore, FoxTPI showed other conserved fragments and well-described residues as the region 206-YGGS-209 (●) (amino acid number corresponding to FoxTIM sequence from *F. oxysporum*), amino acids involved in the correct positioning of the substrate. It has even been reported that this motif (YGGS) interacts with loop 6 (catalytic loop) that functions as a lid that opens and closes to exclude water molecules from the active site, facilitating enzymatic catalysis (Figure 2A) [39]. Another conserved fragments of TPIs is located in loop 3, which are amino acids that allow interaction between monomers to give rise to the catalytically active homodimer. In the FoxTPI sequence, the amino acid residues corresponding to loop 3 are from 60 to 79 (Figure 2). Finally, the FoxTPI enzyme has three cysteine (Cys) residues in its sequence in the positions 125, 126, and 215 (#). It is important to note that the FoxTPI sequence only contains one conserved (Cys 125), while the other two Cys residues (126 and 215) are not conserved with other TPI sequences (Figure 2A). From the alignment of the FoxTPI protein and the identification of these biologically important regions in the sequence of the FoxTPI protein from *F. oxysporum*, it was inferred that this enzyme shows the general characteristics that would allow it to perform its main enzymatic function and that can be used as a target, similarly to other previously characterized TPIs.

The secondary structure motifs of the FoxTPI protein were predicted with PDBsum [43] where a total of eight α-helices and eight β-sheets (Figure 2B) with an overall folding, “(β/α)β(β/α)_6_”, similar to other TPI barrel foldings.

### 3.3. Expression and Purification of FoxTPI

**Figure 3:**
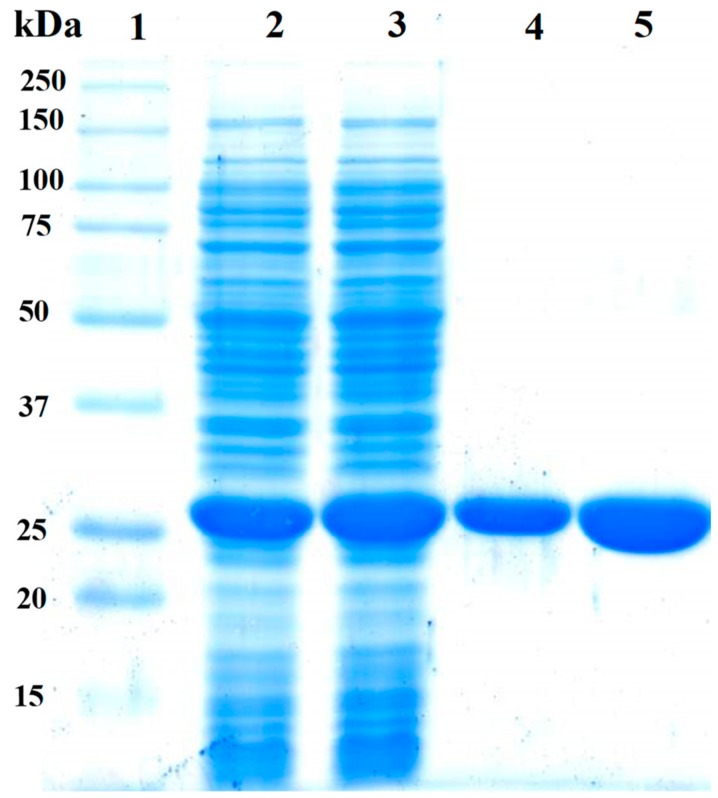
Expression and purification of the recombinant FoxTPI protein analyzed on 12% SDS-PAGE. Lane 1: Marker Precision Plus Protein Kaleidoscope Standards (Bio-Rad, Hercules, CA, USA). Lane 2, Lysate of *E. coli* BL21(DE3)pLyS cells. Lane 3, crude extract of *E. coli* BL21(DE3)pLyS cells. Lanes 4 and 5, correspond to 10 µg and 20 µg, respectively, of the purified FoxTPI. SDS-PAGE gel was stained with colloidal coomassie brilliant blue (R-250) (Sigma-Aldrich, St. Louis, MO, USA).

The pET-3a-HISTEVP-*Fox*TPI vector constructed was used to express the recombinant protein (FoxTPI) in *E*. *coli* BL21 pLyS cells (DE3) under the conditions described in the Materials and Methods section. The recombinant FoxTPI protein was purified by the Ni Sepharose high performance affinity column (GE Healthcare) due, which are contained fused to an N-terminal His tag—the FoxTPI. The His tag was removed using the site-specific protease HisTEVP as previously reported [27]. Purified FoxTPI protein was analyzed SDS-PAGE gel, and a single band of approximately 27 kDa was observed (Figure 3; lane 4). To verify purity and rule out possible contamination with other proteins of purified FoxTPI, we loaded 20 µg of total protein in the same gel (Figure 3; lane 5), and only one band was observed (Figure 3; lane 5). Additionally, in the purification process, we obtained 32 mg of total protein per liter of culture with a specific activity of 5531 µmol·min^−1^·mg^−1^. The purity and concentration of purified enzyme allowed us to biochemically and functionally characterize the protein from *F. oxysporum.*

### 3.4. Characterization of Functional Parameters of the FoxTPI Protein

#### 3.4.1. Oligomeric Status of the FoxTPI Protein

**Figure 4:**
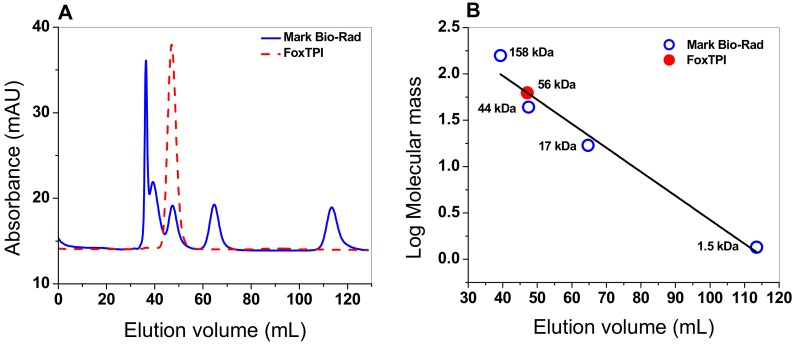
Oligomeric status of the recombinant FoxTPI protein. (**A**) size exclusion chromatography of FoxTPI and gel filtration standard from BioRad, containing thyroglobulin, γ-globulin, ovalbumin, and vitamin B12. The red line represents the recombinant FoxTPI; (**B**) calibration curve of marker proteins drawn with elution volumes versus log of molecular mass (MW) of Bio-Rad’s gel filtration standard (blue spots). The MW of FoxTPI (red circle) is shown on the straight line obtained.

The oligomeric status of FoxTPI protein in solution was determined using gel filtration chromatography. As seen in Figure 4A, a single peak with an elution volume of 47 mL was observed. From the chromatogram obtained for the markers (gel filtration standard # 151-1901 from Bio-Rad), we found that protein mass of the FoxTPI protein corresponds to the native dimers with a relative molecular weigth (MW) of 56 kDa (Figure 4B), and which is in accordance with the MW expected from the amino acid sequence (27,000 × 2 = 54 kDa), whereas the sodium dodecyl sulfate-polyacrylamide gel electrophoresis (SDS-PAGE) analysis revealed a unique band of around 27 kDa (Figure 3). This result is consistent with those reported for other TPIs, such as of *Saccharomyces cerevisiae* [44], and parasites as *Entamoeba histolytica* (EhTPI), *Trypanosoma brucei* (TbTPI), *T*. *cruzy* (TcTPI), and *Encephalitozoon intestinalis* (EiTPI), which were reported with a molecular mass around 27 kDa [45,46,47,48].

#### 3.4.2. Effect of pH and Temperature on the FoxTPI Activity

**Figure 5:**
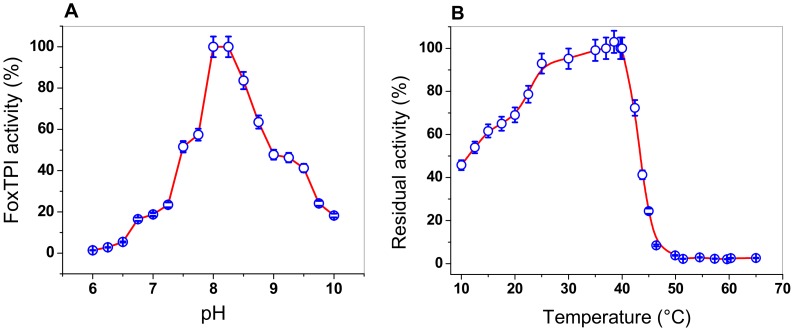
Determination of pH and temperature on the activity of FoxTPI. (**A**) effect of pH on the activity of the FoxTPI protein. The activities were tested by measuring the specific activity under standard conditions at each pH value. The residual activity was tested by measuring the activity under standard conditions at each pH value. The buffers used are described in Materials and Methods; (**B**) the effect of temperature on the activity of FoxTPI protein. The protein was incubated at different temperatures (from 10 to 65 °C) for 20 min in 50 mM Tris buffer at pH 8.0 and the residual activity was measured. In both assays, the data represent the mean ± SD from three independent measurements.

The effect of pH on the activity of FoxTPI from *F. oxysporum* was determined by measuring the specific activity at different pH values, ranging from 6.0 to 10.0. The pH curve obtained shows the classical bell-shaped behavior, with an optimal pH noted at 8.2 (Figure 5A). As seen in Figure 5A, at a pH value of 6.0, no activity was detected for the FoxTPI enzyme, while that at pH value of 7.0, FoxTPI activity was detected around to 20%. However, from pH values from 7.0 to 8.0, the enzyme reached a maximum activity at pH value of 8.0, and the maximum activity was stable at a pH value of 8.25. Furthermore, the FoxTPI enzyme presented a relative activity of 50% at a pH of 9.0, while, for pH values from 9.0 to 10.0, the enzyme activity decreased by around 20%. The optimal pH found for the FoxTPI enzyme (pH value of 8.0) agrees with some reported TPIs; for example, the *Leshmania donovani* TPI recombinant enzyme was stable over the pH range 7.2–9.0 with >90% activity, while *L. mexicana* TPI also appeared to be equally stable over a wide pH range (6.5–8.5), while the pH optimal reported for the recombinant TPI from *Mycoplasma gallisepticum* (rMGTPIA) was 7.6 with a wider pH zone ranging from pH 7.0 to 8.0 [49].

Moreover, we performed assays to evaluate the effect of temperature on the activity of the FoxTPI protein, the enzyme was incubated at different temperatures (10 to 60 °C), and the residual activity was measured as previously reported [18]. As seen in Figure 5B, the FoxTPI protein showed a maximum activity from 35 to 40 °C, and then sharply dropped between 40 and 46 °C. Finally, from 50 to 65 °C, the enzyme lost 100% of its activity. However, when the FoxTPI enzyme was incubated at temperatures below 35 °C, we observed that the FoxTPI enzyme activity slowly decreased between 35 and 25 °C and loses about 55% of its activity at 10 °C (Figure 5B). These results indicate that the enzyme is stable in a range of temperature from 25 to 35 °C. These results agree with those previously reported for the recombinant TPI from *M. gallisepticum* (rMGTPIA), where an optimal temperature of 30 °C was determined [49]. According to the above, the rest of the functional studies with the FoxTPI enzyme were performed in pH value of 7.4 and incubated at a temperature of 37 °C.

#### 3.4.3. Determination of Kinetic Parameters

**Figure 6:**
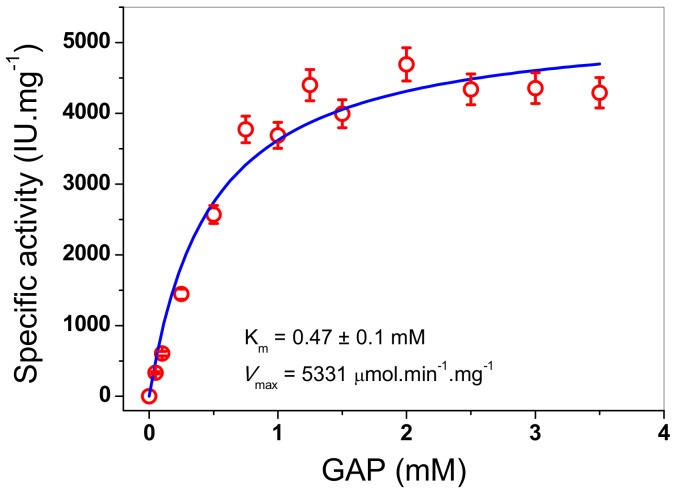
Determination of pH and temperature on the activity of FoxTPI. The effect of pH on the activity of kinetic plots of the FoxTPI determined with GAP. Initial velocity data were obtained varying substrate concentration, indicated in the abscissa axis, and fitted to the Michaelis-Menten equation. The data represent mean ± SD from three independent experiments.

TPI enzymes from diverse organisms have been characterized, which have shown high catalytic activities in the reaction reversible of isomerization of the ketose dihydroxyacetone phosphate (DHAP) to aldose (D)-glyceraldehyde-3-phosphate (GAP). In this work, the kinetic parameters values for the recombinant FoxTPI enzyme were obtained using GAP as the substrate. The initial velocities of the FoxTPI enzyme were obtained at different substrate concentrations and fitted to the Michaelis-Menten equation by nonlinear regression calculations. Figure 6 shows that the FoxTPI has a canonical Michaelis-Menten saturation curve and shows affinity for GAP. Thus, we estimated the kinetic parameters: V_max_ was 5331 μmol min^−1^ mg^−1^, and K_m_ was 0.47 ± 0.1 mM. Interestingly, the data of K_m_ and k_cat_ values (2.9 × 10^5^ min^−1^) obtained for FoxTPI are consistent with data reported in studies involving TPIs from other species (Table 1).

### 3.5. Spectroscopic Characterization of the FoxTPI Protein

#### 3.5.1. Circular Dichroism (CD) Analysis

**Figure 7:**
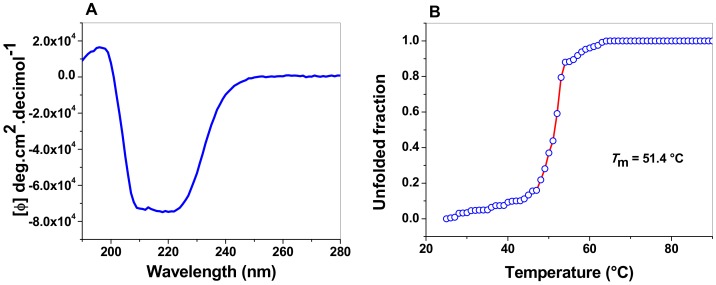
Spectroscopic properties of FoxTPI. (**A**) Far-UV CD spectra of the FoxTPI protein. The protein concentration was 0.4 mg/mL in TE buffer at 25 °C, and the experiments were performed in triplicate. The spectra of blanks were subtracted from those that contained the protein; (**B**) thermal denaturation of FoxTPI. The data are the mean of at least three independent experiments.

A CD analysis in the far UV region (190–260 nm) was used to evaluate the structural characteristics and folding properties of diverse proteins. Figure 7A shows the FoxTPI spectrum; we observed maximum negative absorption peaks at 208 and 222 nm, which is characteristic of the α-helices and β-strands structure of the protein. The result obtained show that the FoxTPI protein is a well-folded protein with a secondary structure like that of a canonical TPI barrel. It is important to mention that the intensity pattern in the far UV region shown for FoxTPI is similar to to other secondary structures of TPIs previously purified and characterized, as in *G. lamblia* (GlTPI) [17], human (HuTPI) [50], *Trichomonas vaginalis* (TvTPI) [55], *Saccharomyces cerevisiae* (TPI) [44], and *Encephalitozoon intestinalis* (EiTPI) [48], between which no significant differences were observed.

#### 3.5.2. Thermal Stability of the FoxTPI Protein

The global thermal stability of the FoxTPI protein was evaluated by ellipticity changes in the structure assessed by CD. A temperature range of 20 to 90 °C was used, and, during the run (temperature increase), the signal was monitored at 222 nm. The temperature increases induced denaturation of the protein. In the denaturation profile, the protein remained at its baseline (native structure) until approximately 43 °C (Figure 7B) and, above this temperature, the enzyme was denatured cooperatively, completing the process at approximately 60 °C. The calculated *T*_m_ was 51.4 °C, which is slightly inferior to the values reported for other TPI enzymes previously characterized from *E. intestinalis* (EiTPI; 52.8 °C) [48], *T. cruzi* (TcTPI; 57.3 °C) [52], *Entamoeba histolytica* (EhTPI; 58.6 °C) [59], *G. lamblia* (GlTPI; 57.5 °C) [17], and *Homo sapiens* (HsTPI; 61.2 °C) [50].

### 3.6. Assay Stability with Guanidine Hydrochloride (Gdn-HCl)

**Figure 8:**
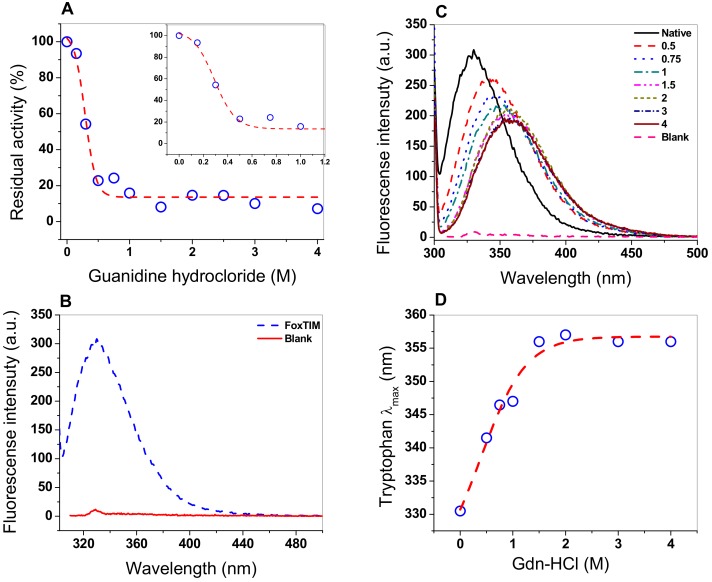
Stability analysis of FoxTPI protein in the presence of Gdn-HCl. (**A**) effect of the Gdn-HCl on the activity of FoxTPI; (**B**) fluorescence emission spectra of the native FoxTPI enzyme; (**C**) intrinsic fluorescence analysis of FoxTPI at various concentrations of Gdn-HCl; (**D**) maximum emission intrinsic fluorescence of the tryptophan/monomers produced by the denaturing induced by Gnd-HCl. Intrinsic fluorescence was obtained by subtracting the blank from the values of total fluorescence intensity. In all the assays, the protein was incubated at 0.2 mg/mL in Tris buffer (50 mM, pH 7.4), in the presence of the indicated concentrations of Gdn-HCl for 2 h at 37 °C; subsequently, the enzymatic activity and fluorescence intensity were obtained. All the experiments were performed in triplicate.

Chaotropic agents, including guanidine hydrochloride, are commonly used as chemical denaturing agents to study the stability and/or denaturation of proteins, conformation, unfolding, and refolding because the denaturing agent alters the tertiary structure of the protein [34]. Therefore, we decided to evaluate the effect of Gdn-HCl on FoxTPI, performing inactivation assays using different concentrations of the compound (Figure 8A), where a sigmoidal behavior of inhibitory activity of FoxTPI was observed with a C_50_ value of 0.28 M, while, at 0.2 M of Gdn-HCl, there was no effect on the inhibitory activity of FoxTPI, whereas, at 0.5 M, activity was practically lost completely.

### 3.7. Structural Analysis through Intrinsic Fluorescence

Fluorescence experiments provide information about the molecular environment of fluorophore molecules. The fluorescence of proteins is generally predominated by tryptophan residudes, so its fluorescence spectrum provides a sensitive tool for the characterization of proteins and their conformation. The intrinsic fluorescence of the tryptophan residues presents in FoxTPI (four by monomers) were analyzed to determine structural changes when the FoxTPI was exposed to Gdn-HCl. The intrinsic fluorescence for the native FoxTPI protein showed a peak at 330 nm with a maximum intensity of 308 arbitrary units (a.u.) (Figure 8B). However, when the protein was exposed in increasing concentrations of Gdn-HCl (0 to 4 M), the fluorescence intensity decreased, until reaching a maximum intensity of 191 a.u (Figure 8C). Moreover, the increase in Gdn-HCl concentration also caused changes in the maximum emission length (λ_max_) of FoxTPI, as seen in Figure 8D. It was observed that the maximum emission of the native enzyme was 330 nm after excitation at 295 nm, which suggests that tryptophan residues were hidden from the hydrophobic environment. Howeover, when the FoxTPI protein was exposed to different concentrations of Gdn-HCl, changes in the intensity of intrinsic fluorescence were again observed due to an increase in redshift; this was caused by changes in the tertiary structure of FoxTPI, exposing the tryptophan residues in the hydrophobic environment. These changes began to be observed from the lowest concentration evaluated (0.5 M); with the increase in denaturant concentration, a displacement of the redshift of 330 to 357 nm occurred, which became constant at a concentration greater than or equal to 1.5 M of Gdn-HCl, indicating an almost complete unfolding of the FoxTPI protein.

### 3.8. Homology Modeling of the FoxTPI Protein

According to a BlastP against Protein Data Bank (PDB), the FoxTPI sequence has an identity of 68.5% with TIM63 (PDB entry 6NEE; [36]), 60.4% with the TPI from *Arabidopsis thaliana* (PDB entry 4OBT; [51]) and 57.8% with *Opisthorchis viverrini* (PDB entry 5ZG4; [60]. Thus, the crystal structure of TIM63 was used to generate the FoxTPI model using the Swiss-Model server [39].

**Figure 9:**
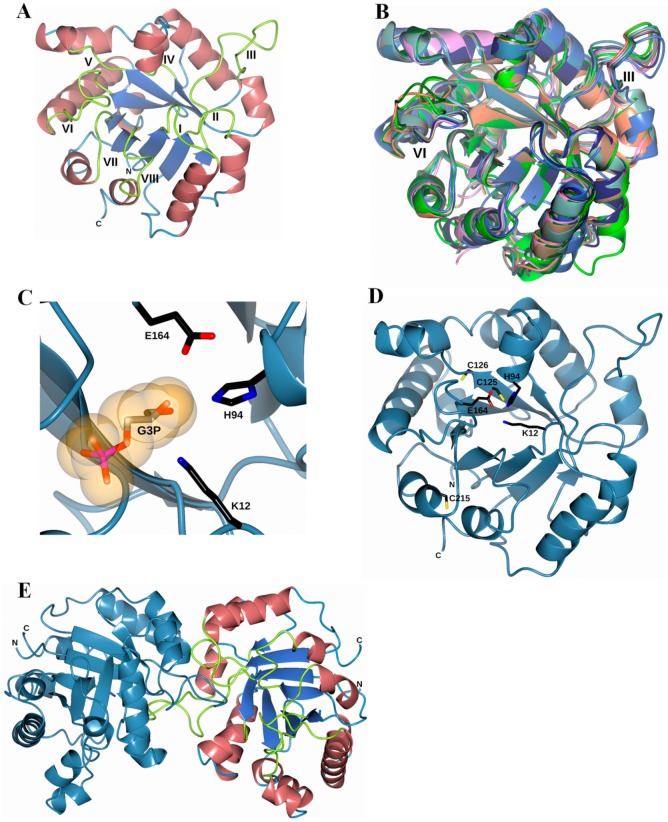
Homology model of the FoxTPI. (**A**) homology model of the FoxTPI showing the typical (βα)_8_-barrel fold; α-helices are shown in Indian red, β-strands are shown in royal blue, and the connecting loops (I-VIII) are shown in greenish-yellow; (**B**) structural superposition of the FoxTPI model (steel-blue) with eight ‘‘closed’’ TPI structures: TmTPI (*T. molitor*; PDB entry 2I9E; coral), AtTPI (*A. thaliana*; PDB entry 4OBT; dark green), CeTPI (*C. elegans;* PDB entry 1MO0; pink), TcTPI (*T. cruzi*; PDB entry 1TCD; sea green), LmTPI (*L. mexicana*; PDB entry 1AMK; pale brown), EhTPI (*E. histolytica*; PDB entry 1M6J; lime), GlTPI (*G. lamblia*; PDB entry 4BI7; midnight blue) and HpTPI (*H. pylori*; PDB entry 2JGQ; royal blue). Note that some structural changes occur within loops III and VI, as indicated; (**C**) active-site region of the FoxTPI model showing the three catalytic residues, Lys12, His94, and Glu164, as black cylinders. The G3P molecule (PDB entry 3UWU) is drawn as orange molecular surface representation; (**D**) representation of the three cysteine residues, Cys125, Cys126, and Cys215 (black cylinders) in the FoxTPI model (steel blue); (**E**) dimeric structure of the FoxTPI model was built with the SWISS-MODEL [39]. In one monomer, the enzyme is colored as in Figure 9A and the other monomer is steel blue.

The superposition of eight “closed” TPI structures is displayed in Figure 9B. These proteins share a similar TPI barrel fold and, in our model, we observed that in both loop III and loop VI, there were major differences when making the structural superposition with other TPIs. The substrate-binding pocket of FoxTPI is created by three key residues: Lys12, His94, and Glu164, in a similar conformation to that of other TPIs (Figure 9C). In addition, the FoxTPI enzyme has three Cys in its amino acid sequence in the positions 125, 126, and 215. In our model, we observed that, according to its position, Cys125 in FoxTPI is the only one that shows equivalence with other Cys of other TPI enzymes (Figure 9D). For example, Cys 125 of FoxTPI (127 in most TPIs) is a strictly conserved amino acid, and it has been proposed that it is related to the correct folding of TPIs [44]. Finally, a dimeric structure of the FoxTPI model was built with SWISS-MODEL (Figure 9E) because the oligomeric status of FoxTPI protein in solution determined by gel filtration chromatography corresponds to the native dimers with a relative molecular weigth (MW) of 56 kDa.

### 3.9. Inactivation of FoxTPI with Proton-Pump Inhibitor Derivatives

Recently, it has been reported that proton-pump inhibitor derivatives exhibit activity against the TPI from *G. lamblia*, through the derivatization of Cys residues. These studies have shown that the chemical derivatization of Cys residues by probes reactive with sulfydryl groups caused important alterations of the enzyme at the functional and at the structural level [18,48]. In addition, it is important to note that the FoxTPI sequence from *F. oxysporum* only contains one conserved (Cys125), while the other two Cys residues (126 and 215) are not conserved with other TPI sequences (Figure 2A).

**Figure 10:**
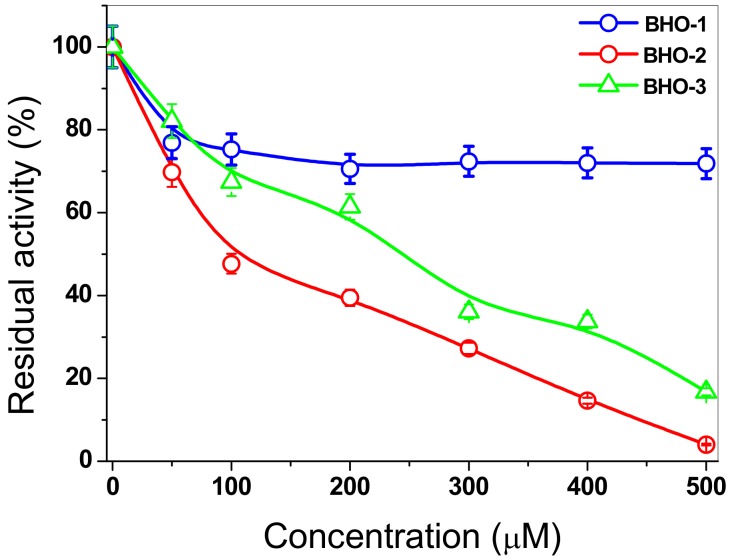
Inactivation assays for the FoxTPI with BHO1, BHO2, and BHO3 compunds. The enzyme was incubated at increasing concentrations of compounds. Afer 2 h of incubation at 37 °C, an aliquot was withdrawn, and the residual activity was determinated as described in the Materials and Methods section.

Based on the above, in this work, we estudied the inactivation of FoxTPI via the derivatization of its Cys residues in order to investigate the protein’s potential as a target for rational drug design for the control of pathogenic fungi as *F*. *oxysporum*. For this purpose, we evaluated the effect of three compounds (with proton pump inhibitory scaffolding), which demonstrated an inhibitory effect on the TPI of *G*. *lamblia* [18]. These same compounds (BHO1, BHO2, BHO3) were tested to assess their effect on the activity of the recombinant FoxTPI enzyme using increasing concentrations. In the case of inactivation of FoxTPI by BHO1, a 20% decrease in FoxTPI activity was observed at 50 μM: while, for that from 100 to 500 µM of this compound, a decrease of 30% was observed (Figure 10). However, the results obtained with the compounds BHO2 and BOH3, the inactivation process was different for both compounds, where 50% enzymatic inhibition was observed at 92 and 241 µM concentrations, respectively, and, at higher concentrations (500 µM), the FoxTPI protein was completely inactivated. These results indicate that FoxTPI enzyme from *F. oxysporum* can be inactivated through the derivatization of its Cys residues. However, these IC_50_ values determined for the FoxTPI enzyme are in disagreement with the previously reported for these same compounds in the GlTIM from *G. lamblia* [18], where IC_50_ lower values were reported. On the other hand, based on homology modeling of the FoxTPI protein, we consider that the Cys125 residue could be responsible for the loss of activity because the Cys215 residue in FoxTPI enzyme is located in non-conserved sites, and it is more exposed to the solvent (Figure 9D). This is of great relevance because previous studies have studied and proposed it as a potential target to the TPI of various pathogenic organisms for rational drug design [18,20]. According to the results obtained, we have demonstrated that FoxTPI can be totally and efficiently inactivated through the derivatization of Cys residues. Even when relating the data and analyzing the alignment made, this enzyme could be a good target for the design of molecules in this fungus because the compounds can act on this protein and have physiological and metabolic effects on the fungus.

## 4. Conclusions

In this study, the recombinant TPI of *F*. *oxysporum* was isolated and cloned for the first time, being the only species of *Fusarium* to which the structural and functional characterization of the TPI enzyme has been performed. Interestingly, the bacterial expression system used was very efficient, and, together with the purification system (affinity) used, a good purity (around 97%) of the recombinant protein was achieved, which allowed us to obtain its kinetic values, such as *Km*, *Vmax*, and other biochemical parameters. These results also enable a better understanding of FoxTPI, which can be used in future studies for the design of new molecules of this and/or other pathogenic fungi due to its relevance in the medical and agricultural area.

## Figures and Tables

**Table 1:** Steady-state kinetic parameters of the TPI from *F*. *oxysporum* and other TPIs previously reported.

Organism	k_cat_ (GAP) (min^−1^)	K_m_ (GAP) (mM)	k_cat_/K_m_ (GAP) (min^−1^ mM^−1^)	Reference
*Fusarium oxysporum*	2.9 × 10^5^	0.47	6.0 × 10^5^	[This study]
*Encephalitozoon intestinalis*	1.0 × 10^5^	0.83	2.0 × 10^6^	[48]
*Saccharomyces cerevisiae*	2.8 × 10^5^	1.10	4.3 × 10^6^	[44]
*Homo sapiens*	1.8 × 10^5^	0.74	4.2 × 10^6^	[50]
*Giardia lamblia*	4.6 × 10^5^	0.78	9.8 × 10^6^	[17]
*Arabidopsis thaliana*	1.5 × 10^5^	0.48	5.3 × 10^6^	[51]
*Trypanozoma cruzi*	2.7 × 10^5^	0.43	1.0 × 10^7^	[52]
*Leishmania mexicana*	2.5 × 105	0.41	1.0 × 10^7^	[53]
*Plasmodium falciparum*	2.5 × 10^5^	0.35	7.1 × 10^5^	[54]
*Trichomonas vaginalis*	7.9 × 10^4^	0.23	3.5 × 10^5^	[55]
*Vibrio marinus*	4.2 × 10^5^	1.9	2.2 × 10^5^	[56]
*Helicobacter pilory*	8.8 × 10^4^	3.5	2.6 × 10^4^	[57]
*Chicken muscle*	1.9 × 10^5^	0.29	1.1 × 10^7^	[58]

## Supplementary Materials

The following are available online at https://www.mdpi.com/2076-2607/8/1/40/s1, Figure S1: Nucleotide sequences of Tpi’s from different Fusarium species used for primers design. FFUJ_03280 (*Fusarium fujikuroi*), FOC1_g10013520 (*F*. *oxysporum* f. sp. cubense race 1), FPRO_05057 (*Fusarium proliferatum*), FGRAMPH1_01G22955 (*Fusarium graminearum*), FOMG_04813 (*F*. *oxysporum* f. sp. melonis), FOZG_04985 (*Fusarium oxysporum*), FOXG_02233 (*F*. *oxysporum* f. sp. lycopersici), FOC4_g10010590 (*F*. *oxysporum* f. sp. cubense race 4). The sequences were obtained from FungiDB: The Fungal and Oomycete Genomics Resource (https://fungidb.org/fungidb/). Table S1: List of primers used in this study.
