# Supplementary material for: Gene Cloning, Recombinant Expression, Characterization, and Molecular Modeling of the Glycolytic Enzyme Triosephosphate Isomerase from Fusarium oxysporum
Source: Microorganisms. 2019 Dec 24;8(1):40. doi: 10.3390/microorganisms8010040 (PMC7022633; doi:10.3390/microorganisms8010040)
Supplement: Supplementary file 1 [file microorganisms-08-00040-s001.zip › Supplementary/Figure S1.docx]

FFUJ_03280 ATGGCTCGCAAATTCTTCGTCGGCGGCAACTTCAAGATGAACGGCTCCAAGTCGTCTATCAAGGAGATTGTCGATAACCTCAACAACGCTGATCTCGACAAGAACGCTGAGGTTGTTGTCTCTCCTCCTGCCATCTACCT

FOC1_g10013520 ATGGCTCGCAAGTTCTTCGTCGGCGGCAACTTCAAGATGAACGGCTCCAAGTCGTCCATCAAGGAGATTGTCGATAACCTCAACAACGCTGATCTCGACAAGAACGCTGAGGTCGTTGTCTCTCCTCCTGCCATCTACCT

FPRO_05057 ATGGCTCGCAAGTTCTTCGTCGGCGGCAACTTCAAGATGAACGGCTCCAAGTCGTCTATCAAGGAGATTGTCGATAACCTCAACAACGCTGATCTCGACAAGAACGCTGAGGTTGTTGTCTCTCCTCCTGCCATCTACCT

FGRAMPH1_01G22955 ATGGCTCGCAAGTTCTTCGTCGGCGGCAACTTCAAGATGAACGGCTCCAAGTCGTCCATCAAGGAGATTGTCGAGAACCTCAACAACGCTGACCTTGACAAGAACGCTGAGGTCGTTGTCTCCCCTCCTGCCCTCTACCT

FOMG_04813 ATGGCTCGCAAGTTCTTCGTCGGCGGCAACTTCAAGATGAACGGCTCCAAGTCGTCCATCAAGGAGATTGTCGATAACCTCAACAACGCTGATCTCGACAAGAACGCTGAGGTCGTTGTCTCTCCTCCTGCCATCTACCT

FOZG_04985 ATGGCTCGCAAGTTCTTCGTCGGCGGCAACTTCAAGATGAACGGCTCCAAGTCGTCCATCAAGGAGATTGTCGATAACCTCAACAACGCTGATCTCGACAAGAACGCTGAGGTCGTTGTATCTCCTCCTGCCATCTACCT

FOXG_02233 ATGGCTCGCAAGTTCTTCGTCGGCGGCAACTTCAAGATGAACGGCTCCAAGTCGTCCATCAAGGAGATTGTCGATAACCTCAACAACGCTGATCTCGACAAGAACGCTGAGGTCGTTGTCTCTCCTCCTGCCATCTACCT

FOC4_g10010590 ATGGCTCGCAAGTTCTTCGTCGGCGGCAACTTCAAGATGAACGGCTCCAAGTCGTCCATCAAGGAGATTGTCGATAACCTCAACAACGCTGATCTCGACAAGAACGCTGAGGTCGTTGTCTCTCCTCCTGCCATCTACCT

*********** ******************************************** ***************** ***************** ** ***************** ***** ** ********* *******

FFUJ_03280 CCCTCTCGTCCGTGAGACCCTCCGCAAGGACATCGAGGTCGCCGCCCAGAACGTCTTCAACAAGCCCAACGGTGCCTTCACCGGCGAGATCTCCGTCTCCCAGCTCAAGGATAGCGACATCAACTGGGTCATCCTCGGTC

FOC1_g10013520 CCCTCTCGTCCGTGAGACCCTCCGCAAGGACATCGAGGTCGCCGCCCAGAACGTCTTCAACAAGCCCAACGGTGCTTTCACCGGCGAGATCTCCGTCTCCCAGCTCAAGGACAGCGACATCAACTGGGTCATTCTCGGCC

FPRO_05057 CCCTCTCGTCCGTGAGACCCTCCGCAAGGACATCGAGGTCGCCGCCCAGAACGTCTTCAACAAGCCCAACGGTGCCTTCACCGGCGAGATCTCCGTCTCCCAGCTCAAGGATAGCGACATCAACTGGGTCATCCTCGGTC

FGRAMPH1_01G22955 CCCCCTTGTCCGCGAGACTCTCCGCAAGGAAATCGAGGTTGCCGCCCAGAACGTCTACGACAAGCCCAACGGTGCTTTCACTGGCGAGATCTCCGTCTCCCAGCTCAAGGATAGCGATATCAACTGGGCTATCCTCGGCC

FOMG_04813 CCCTCTCGTCCGTGAGACCCTCCGCAAGGACATCGAGGTCGCCGCCCAGAACGTCTTCAACAAGCCCAACGGTGCTTTCACCGGCGAGATCTCCGTCTCCCAGCTCAAGGACAGCGACATCAACTGGGTCATTCTCGGCC

FOZG_04985 CCCTCTCGTCCGTGAGACCCTCCGCAAGGACATCGAGGTCGCCGCCCAGAACGTCTTCAACAAGCCCAACGGTGCTTTCACCGGCGAGATCTCCGTCTCCCAGCTCAAGGACAGCGACATCAACTGGGTCATTCTCGGCC

FOXG_02233 CCCTCTCGTCCGTGAGACCCTCCGCAAGGACATCGAGGTCGCCGCCCAGAACGTCTTCAACAAGCCCAACGGTGCTTTCACCGGCGAGATCTCCGTCTCCCAGCTCAAGGACAGCGACATCAACTGGGTCATTCTCGGCC

FOC4_g10010590 CCCTCTCGTCCGTGAGACCCTCCGCAAGGACATCGAGGTCGCCGCCCAGAACGTCTTCAACAAGCCCAACGGTGCTTTCACCGGCGAGATCTCCGTCTCCCAGCTCAAGGACAGCGACATCAACTGG-------------

*** ** ***** ***** *********** ******** **************** * **************** ***** ***************************** ***** *********

FFUJ_03280 ACTCTGAGCGTCGTGAGATCCTCGGCGAGTCTGACGAGACCATCTCCGCCAAGACCAAGTACGCCACTGAGAACGGCCTCAAGGTCATCTGGTGCTGCGGCGAGACCCTCGAAACCCGTGAGGCTGGCAAGACCATCGAC

FOC1_g10013520 ACTCT------------------------------------------------------------------------------GTCATCTGGTGCTGCGGCGAGACCCTCGAGACCCGTGAGGCTGGCAAGACCATCGAC

FPRO_05057 ACTCTGAGCGTCGTGAGATCCTCGGCGAGTCTGACGAGACCATCTCCGCCAAGACCAAGTACGCCACTGAGAACGGCCTCAAGGTCATCTGGTGCTGCGGCGAGACCCTCGAAACCCGTGAGGCTGGCAAGACCATCGAC

FGRAMPH1_01G22955 ACTCTGAGCGTCGAACCATCATCGGCGAGTCTGATGAGGTCATCTCCTCCAAGACCAAGTACGCCACCGAGAACGGCCTCAAGGTCATCTGGTGCTGCGGCGAGTCCCTCGAGACCCGTGAGGCTGGCAAGACCATTGAG

FOMG_04813 ACTCTGAGCGTCGTGAGATTCTCGGCGAGTCTGACGAGACGATCTCCTCCAAGACCAAGTACGCCACTGAGAACGGCCTCAAGGTCATCTGGTGCTGCGGCGAGACCCTCGAGACCCGTGAGGCTGGCAAGACCATCGAC

FOZG_04985 ACTCTGAGCGTCGTGAGATTCTCGGCGAGTCTGACGAGACGATCTCCTCCAAGACCAAGTACGCCACTGAGAACGGCCTCAAGGTCATCTGGTGCTGCGGCGAGACCCTCGAGACCCGTGAGGCTGGCAAGACCATCGAC

FOXG_02233 ACTCTGAGCGTCGTGAGATTCTCGGCGAGTCTGACGAGACGATCTCCTCCAAGACCAAGTACGCCACTGAGAACGGCCTCAAGGTCATCTGGTGCTGCGGCGAGACCCTCGAGACCCGTGAGGCTGGCAAGACCATCGAC

FOC4_g10010590 --------CGTCGTGAGATTCTCGGCGAGTCTGACGAGACGATCTCCTCCAAGACCAAGTACGCCACTGAGAACGGCCTCAAGGTCATCTGGTGCTGCGGCGAGACCCTCGAGACCCGTGAGGCTGGCAAGACCATCGAC

********************* ******* *********************** **

FFUJ_03280 TTCGTCTCTAAGCAGCTCGAGTCCCTCAAGTCCCAGATCTCCGACTGGTCCAACATTGTCATTGCCTACGAGCCCATCTGGGCTATCGGCACCGGCAAGGTTGCTACCACTGAGCAGGCTCAGGAGGTCCACAAGGCTAT

FOC1_g10013520 TTCGTCTCTAAGCAGCTCGAGTCCCTCAAGTCCCAGATCTCCGACTGGTCCAACATTGTCATTGCCTACGAGCCCATCTGGGCTATCGGCACTGGCAAGGTTGCTACCACTGAGCAGGCCCAGGAGGTCCACAAGGCTAT

FPRO_05057 TTCGTCTCTAAGCAGCTCGAGTCCCTCAAGTCCCAGATCTCCGACTGGTCCAACCTTGTCATTGCCTACGAGCCCATCTGGGCTATCGGCACCGGCAAGGTTGCTACCACTGAGCAGGCTCAGGAGGTCCACAAGGCTAT

FGRAMPH1_01G22955 TTCGTCTCTGCCCAGATCGAGTCTCTCAAGTCTCAGATCTCCGACTGGTCCAACATTGTCATCGCCTACGAGCCCATCTGGGCCATTGGCACTGGCAAGGTTGCTACCACTGAGCAGGCCCAGGAGGTCCACAAGGCCAT

FOMG_04813 TTCGTCTCTAAGCAGCTCGAGTCCCTCAAGTCCCAGATCTCCGACTGGTCCAACATTGTCATTGCCTACGAGCCCATCTGGGCTATCGGCACTGGCAAGGTTGCTACCACTGAGCAGGCCCAGGAGGTCCACAAGGCTAT

FOZG_04985 TTCGTCTCTAAGCAGCTCGAGTCCCTCAAGTCCCAGATCTCCGACTGGTCCAACATTGTCATTGCCTACGAGCCCATCTGGGCTATCGGCACTGGCAAGGTTGCTACCACTGAGCAGGCCCAGGAGGTCCACAAGGCTAT

FOXG_02233 TTCGTCTCTAAGCAGCTCGAGTCCCTCAAGTCCCAGATCTCCGACTGGTCCAACATTGTCATTGCCTACGAGCCCATCTGGGCTATCGGCACTGGCAAGGTTGCTACCACTGAGCAGGCCCAGGAGGTCCACAAGGCTAT

FOC4_g10010590 TTCGTCTCTAAGCAGCTCGAGTCCCTCAAGTCCCAGATCTCCGACTGGTCCAACATTGTCATTGCCTACGAGCCCATCTGGGCTATCGGCACTGGCAAGGTTGCTACCACTGAGCAGGCCCAGGAGGTCCACAAGGCTAT

********* *** ******* ******** ********************* ******* ******************** ** ***** ************************** ***************** **

FFUJ_03280 CCGCGACCTCCTCCGTGGTATCAGCGACAAGGTTGCTGACGAGACCCGAATCCTCTACGGCGGTAGTGTCAACGAGAAGAACTGCGGCGAGCTCTCCAAGCAGCCCGATATTGACGGTTTCCTTGTTGGCGGTGCTTCTC

FOC1_g10013520 CCGTGACCTCCTCCGTGGTATCAGCGACAAGGTTGCTGACGAGACCCGAATCCTCTACGGCGGCAGTGTTAACGAGAAGAACTGCGGCGAGCTCTCCAAGCAGCCCGACATTGATGGTTTCCTCGTTGGCGGTGCTTCTC

FPRO_05057 CCGTGACCTCCTCCGTGGTATCAGCGACAAGGTTGCTGACGAGACCCGAATCCTCTACGGCGGCAGTGTCAACGAGAAGAACTGCGGCGAGCTCTCCAAGCAGCCCGATATTGACGGTTTCCTTGTTGGCGGTGCTTCTC

FGRAMPH1_01G22955 CCGTGACCTCCTCCGCAGCATCAGCGACAAGGTTGCTGATGAGACCCGCATCCTTTACGGAGGCAGTGTCAACGAGAAGAACTGCGGCGAGCTCTCCAAGCAGGCCGACATTGACGGTTTCCTCGTTGGCGGTGCTTCTC

FOMG_04813 CCGTGACCTCCTCCGTGGTATCAGCGACAAGGTTGCTGACGAGACCCGAATCCTCTACGGCGGCAGTGTTAACGAGAAAAACTGCGGCGAGCTCTCCAAGCAGCCCGACATTGATGGTTTCCTCGTTGGCGGTGCTTCTC

FOZG_04985 CCGTGACCTCCTCCGTGGTATCAGCGACAAGGTTGCTGACGAGACCCGAATCCTCTACGGCGGCAGTGTTAACGAGAAGAACTGCGGCGAGCTCTCCAAGCAGCCCGACATTGATGGTTTCCTCGTTGGCGGTGCTTCTC

FOXG_02233 CCGTGACCTCCTCCGTGGTATCAGCGACAAGGTTGCTGACGAGACCCGAATCCTCTACGGCGGCAGTGTTAACGAGAAAAACTGCGGCGAGCTCTCCAAGCAGCCCGACATTGATGGTTTCCTCGTTGGCGGTGCTTCTC

FOC4_g10010590 CCGTGACCTCCTCCGTGGTATCAGCGACAAGGTTGCTGACGAGACCCGAATCCTCTACGGCGGCAGTGTTAACGAGAAGAACTGCGGCGAGCTCTCCAAGCAGCCCGACATTGATGGTTTCCTCGTTGGCGGTGCTTCTC

*** *********** * ******************** ******** ***** ***** ** ***** ******** ************************ **** ***** ******** ****************

FFUJ_03280 TCAAGCCTGCTTTCGTCGACATCATCAACGCTACCAAGCAGTAA

FOC1_g10013520 TCAAGCCTGCTTTCGTCGACATCATCAATGCTACCAAGCAGTAA

FPRO_05057 TCAAGCCTGCTTTCGTCGACATCATCAACGCTACCAAGCAGTAA

FGRAMPH1_01G22955 TCAAGCCTGCTTTCGTCGACATCATCAACGCTACCAAGCAGTAA

FOMG_04813 TCAAGCCTGCTTTCGTCGACATCATCAACGCTACCAAGCAGTAA

FOZG_04985 TCAAGCCTGCTTTCGTCGACATCATCAACGCTACCAAGCAGTAA

FOXG_02233 TCAAGCCTGCTTTCGTCGACATCATCAACGCTACCAAGCAGTAA

FOC4_g10010590 TCAAGCCTGCTTTCGTCGACATCATCAACGCTACCAAGCAGTAA

**************************** ***************

Figure S1. Nucleotide sequences of Tpi's from different *Fusarium* species used for primers design. FFUJ_03280 (*Fusarium fujikuroi*), FOC1_g10013520 (*F*. *oxysporum* f. sp. cubense race 1), FPRO_05057 (*Fusarium proliferatum*), FGRAMPH1_01G22955 (*Fusarium graminearum*), FOMG_04813 (*F*. *oxysporum* f. sp. melonis), FOZG_04985 (*Fusarium oxysporum*), FOXG_02233 (*F*. *oxysporum* f. sp. lycopersici), FOC4_g10010590 (*F*. *oxysporum* f. sp. cubense race 4).The sequences were obtained from FungiDB: The Fungal and Oomycete Genomics Resource (https://fungidb.org/fungidb/).
