# Supplementary material for: Gene Cloning, Recombinant Expression, Characterization, and Molecular Modeling of the Glycolytic Enzyme Triosephosphate Isomerase from Fusarium oxysporum
Source: Microorganisms. 2019 Dec 24;8(1):40. doi: 10.3390/microorganisms8010040 (PMC7022633; doi:10.3390/microorganisms8010040)
Supplement: Supplementary file 1 [file microorganisms-08-00040-s001.zip › Supplementary/Table S1.docx]

**Supplementary file**

**Table 1.** List of primers used in this study.

| **Strain *E. coli*** | **Relevant Characteristic(s) or Sequence** | **Reference** |
| --- | --- | --- |
| TOP10F´ | F– mcrA ∆(mrr-hsdRMS-mcrBC) φ80lacZ∆M15 ∆lacX74 recA1 araD139 ∆(araA-leu)7697 galU galK rpsL endA1 nupG. | Invitrogen |
| BL21(DE3)pLyS | F^−^ ompT gal dcm lon hsdS_B_(r_B_^−^ m_B_^−^) λ(DE3 [lacI lacUV5-T7 gene 1 ind1 sam7 nin5]). | [Novagen] |
| **Plasmids** |  |  |
| pJET-*FoxTPI* | pJET-*FoxTPI* plasmid carrying the *TPI* gene of *F. oxysporum*, *Amp^R^* | This study |
| pET-3a-HISTEVP-*FoxTPI* | pET-3a-HISTEVP-*FoxTPI* carrying the *TPI* gene of *F. oxysporum* , *Amp^R^* | This study |
| **Oligonucleotides** | **Primer Sequence** |  |
| LSU Fw  LSU Rv  Tpi Fw | 5’- ACCCGCTGAACTTAAGC -3’  5’- TCCTGAGGG AAACTT CG -3’  5- TTAT**CATATG**GCTCGCAAGTTCTTCGT -3′ | [21]  This study |
| Tpi Rv | 5′- AATA**GGATCC**TTACTGCTTGGTAGC(G/A)TTGA -3′ | This study |
| pJet Forward  pJet Reverse | 5’-CGACTCACTATAGGGAGAGCGGC-3’  5’-AAGAACATCGATTTTCCATGGCAG-3’ | This stuy |

The locations of the restriction sites are in bold and underlined.
